# Supplementary figures and images for: Venestatin from parasitic helminths interferes with receptor for advanced glycation end products (RAGE)-mediated immune responses to promote larval migration
Source: PLoS Pathog. 2021 Jun 3;17(6):e1009649. doi: 10.1371/journal.ppat.1009649 (PMC8205142; doi:10.1371/journal.ppat.1009649)

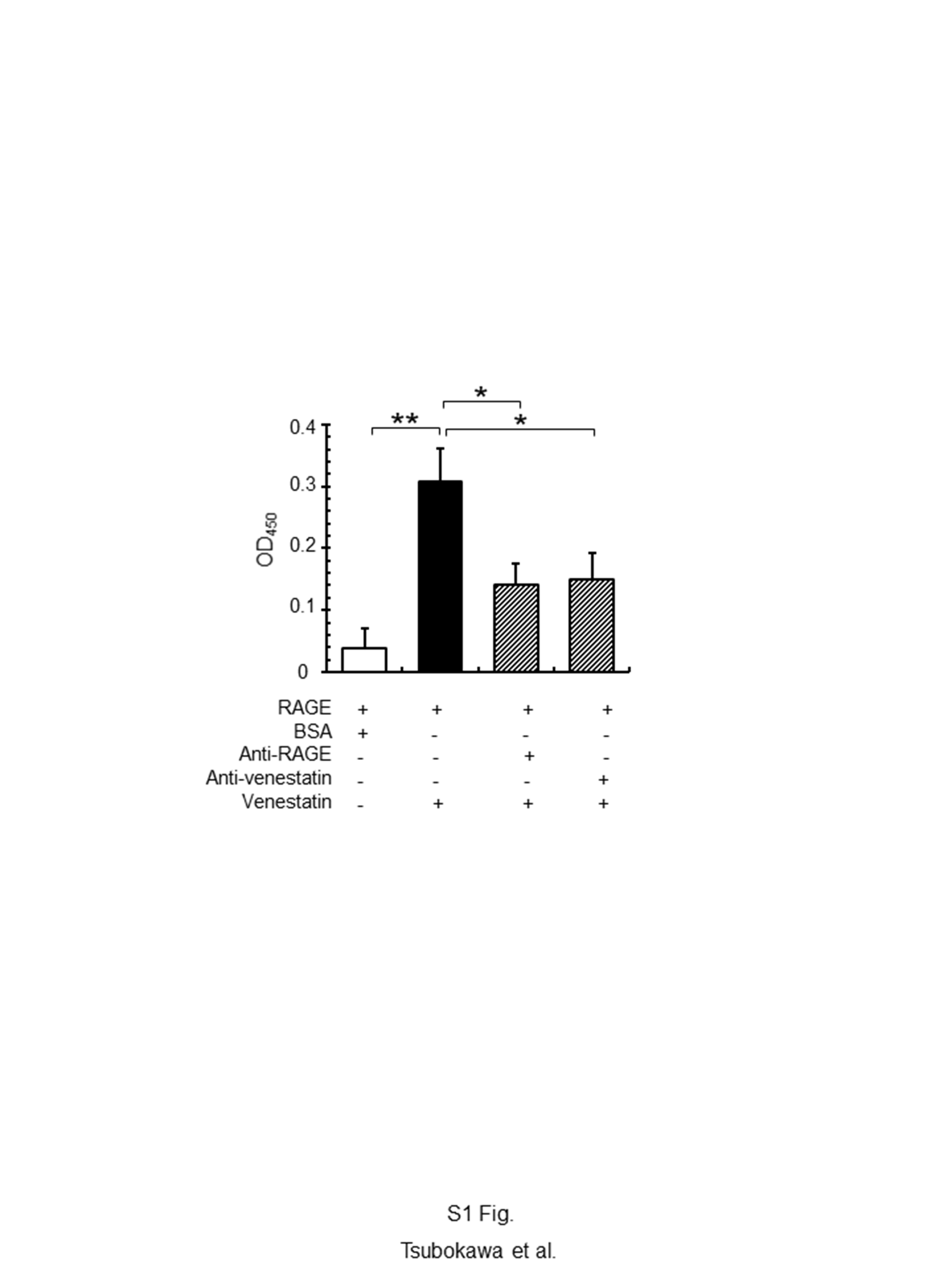

Supplement: S1 Fig — RAGE-coated wells were treated with or without anti-RAGE antibodies. Venestatin was pre-incubated with or without anti-venestatin antibodies and added the wells. After washing, bound venestatin was detected with biotin-labelled anti-venestatin. Data are expressed as means ± SDs of three independent experiments. *p < 0.01; **p < 0.001. (TIF) [file ppat.1009649.s001.TIF]

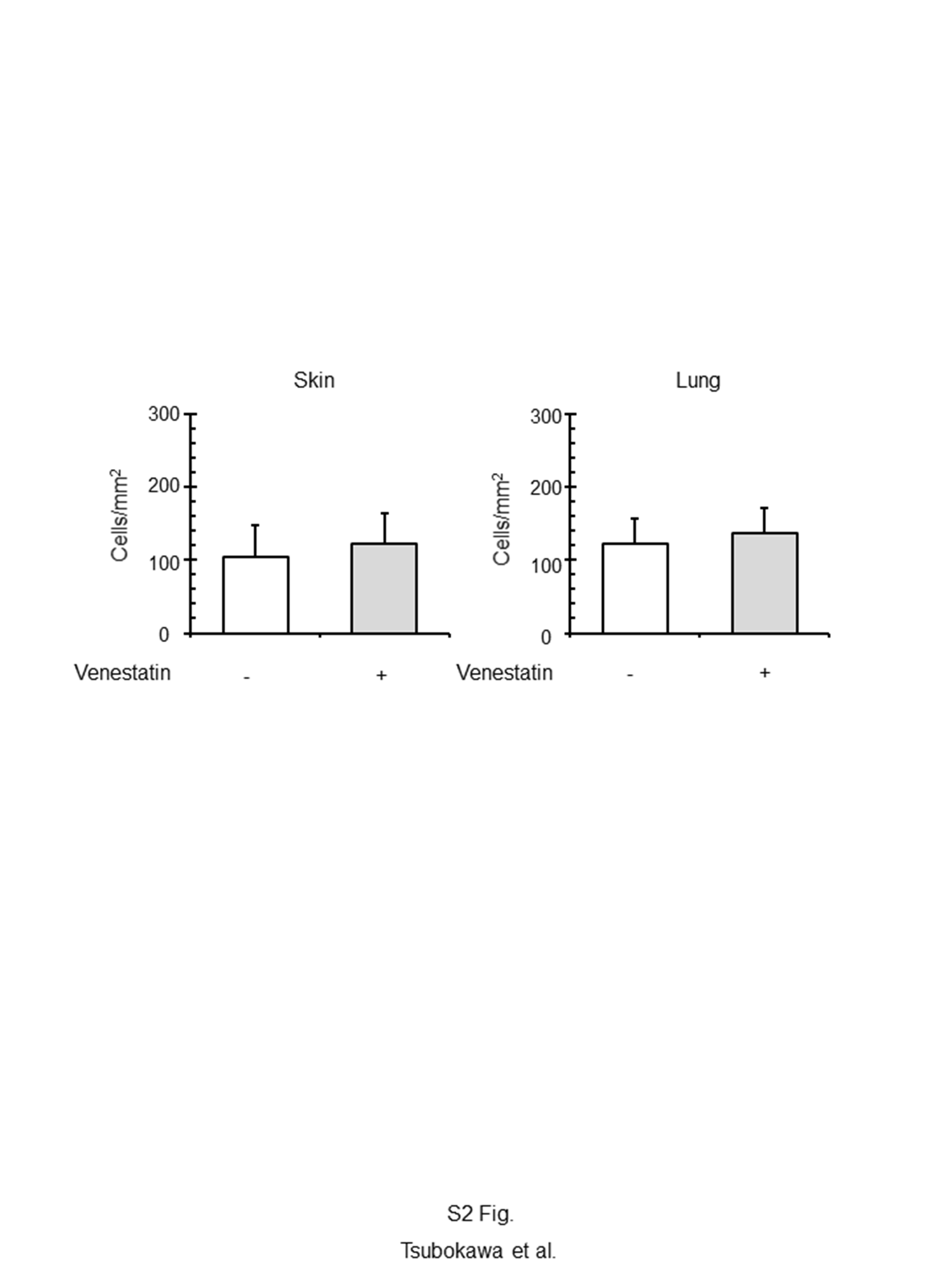

Supplement: S2 Fig — Venestatin or PBS was injected into the hind footpad and instilled intranasally each mouse. After 8 or 48 h, the footpads and lungs were collected, respectively, and sections were stained with H&E. Data are expressed as means ± SDs of 12 fields from two mice. (TIF) [file ppat.1009649.s002.TIF]

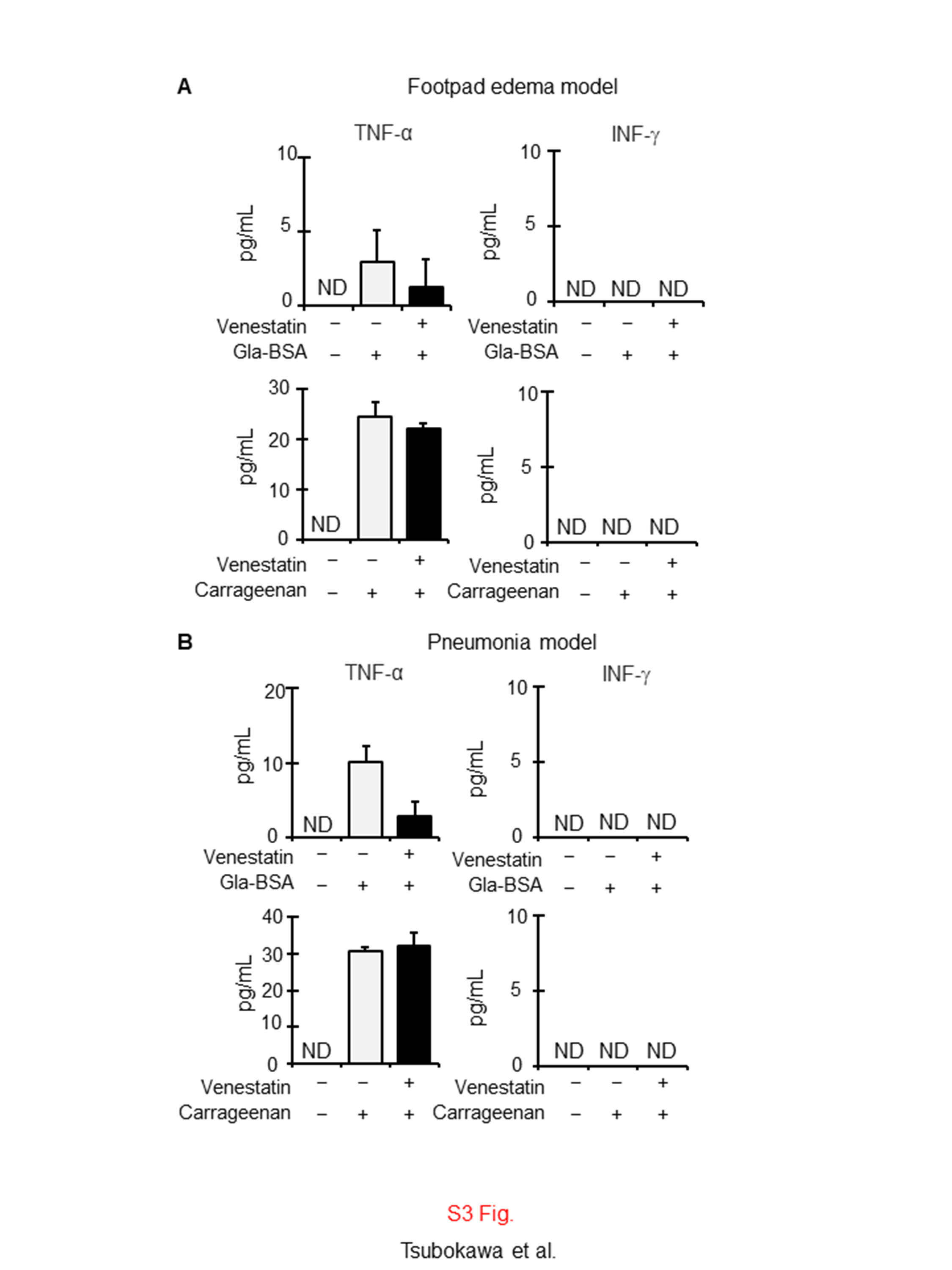

Supplement: S3 Fig — (A) Mouse footpad edema model. (B) Mouse pneumonia model. Concentrations of cytokines in the serum were measured by ELISA. The minimum detectable concentrations of TNF-α and IFN-γ were 3.58 and 2.05 pg/mL, respectively. Data are expressed as means ± SDs of 3 mouse sera. ND, no detection. (TIF) [file ppat.1009649.s003.TIF]

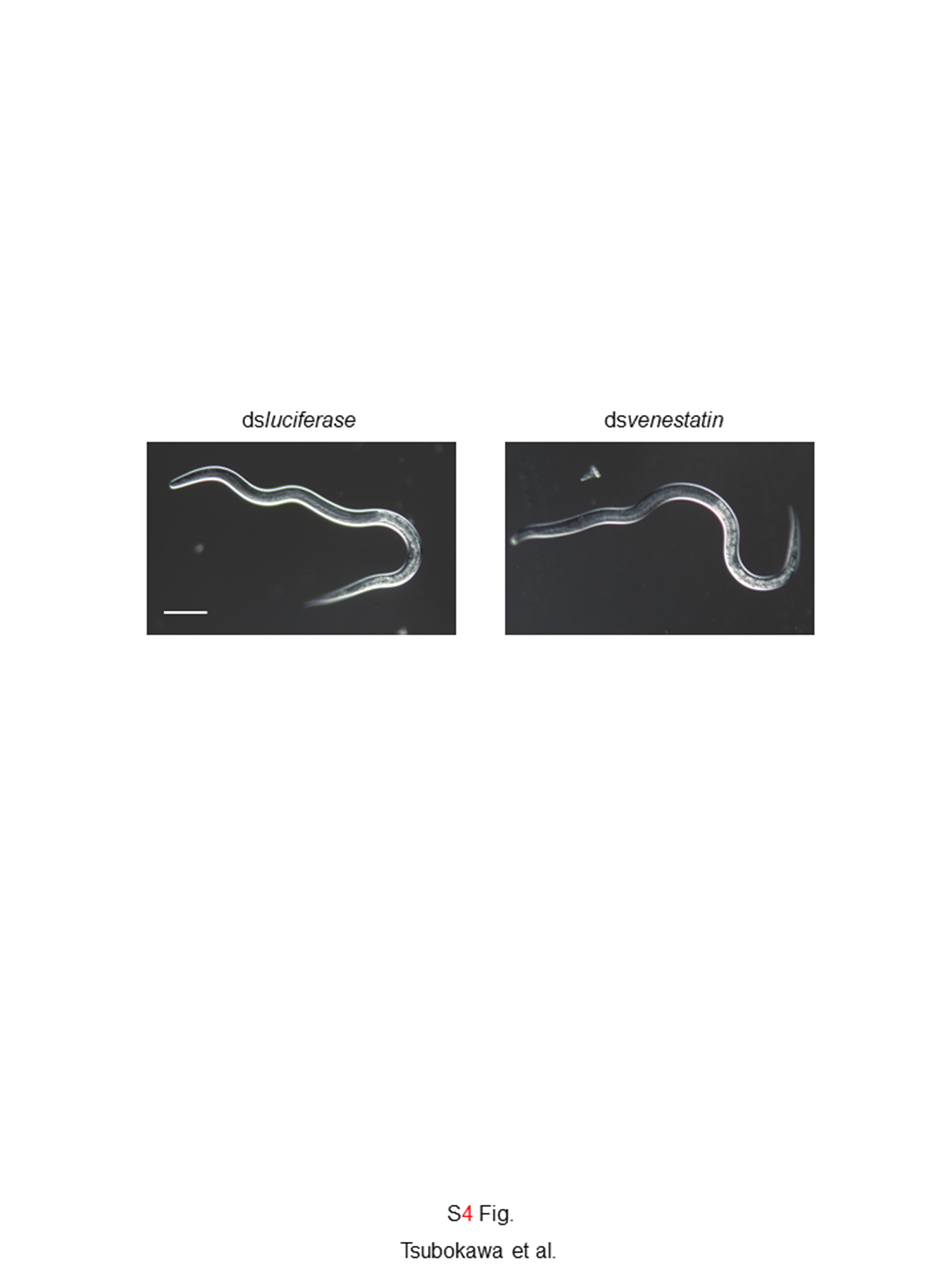

Supplement: S4 Fig — Infective lung stage larvae (LL3s) of S. venezuelensis were incubated with dsluciferase or dsvenestatin. Differential interference contrast (DIC) images of larvae are shown. Scale bar: 50 μm. (TIF) [file ppat.1009649.s004.TIF]

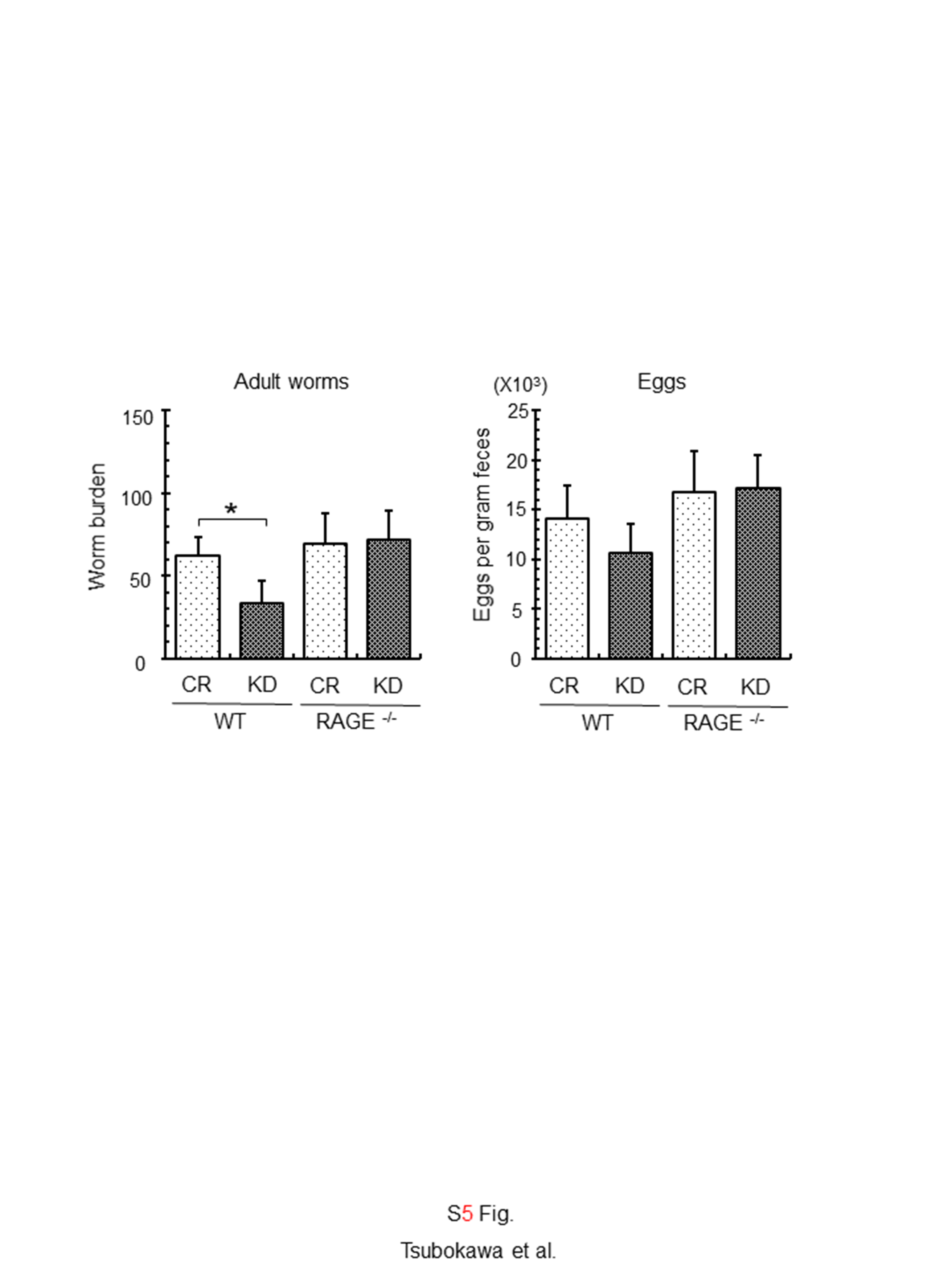

Supplement: S5 Fig — Wild-type (WT) or RAGE-null (RAGE-/-) mice were infected with 2,000 LL3s treated with dsluciferase (CR) or dsvenestatin (KD). Small intestinal adult worm burden and fecal egg output from WT or RAGE-/- mice at day 7 (168 h) p.i. are shown. Data are expressed as means ± SDs of 6 mice from two independent experiments. *p < 0.01. (TIF) [file ppat.1009649.s005.TIF]

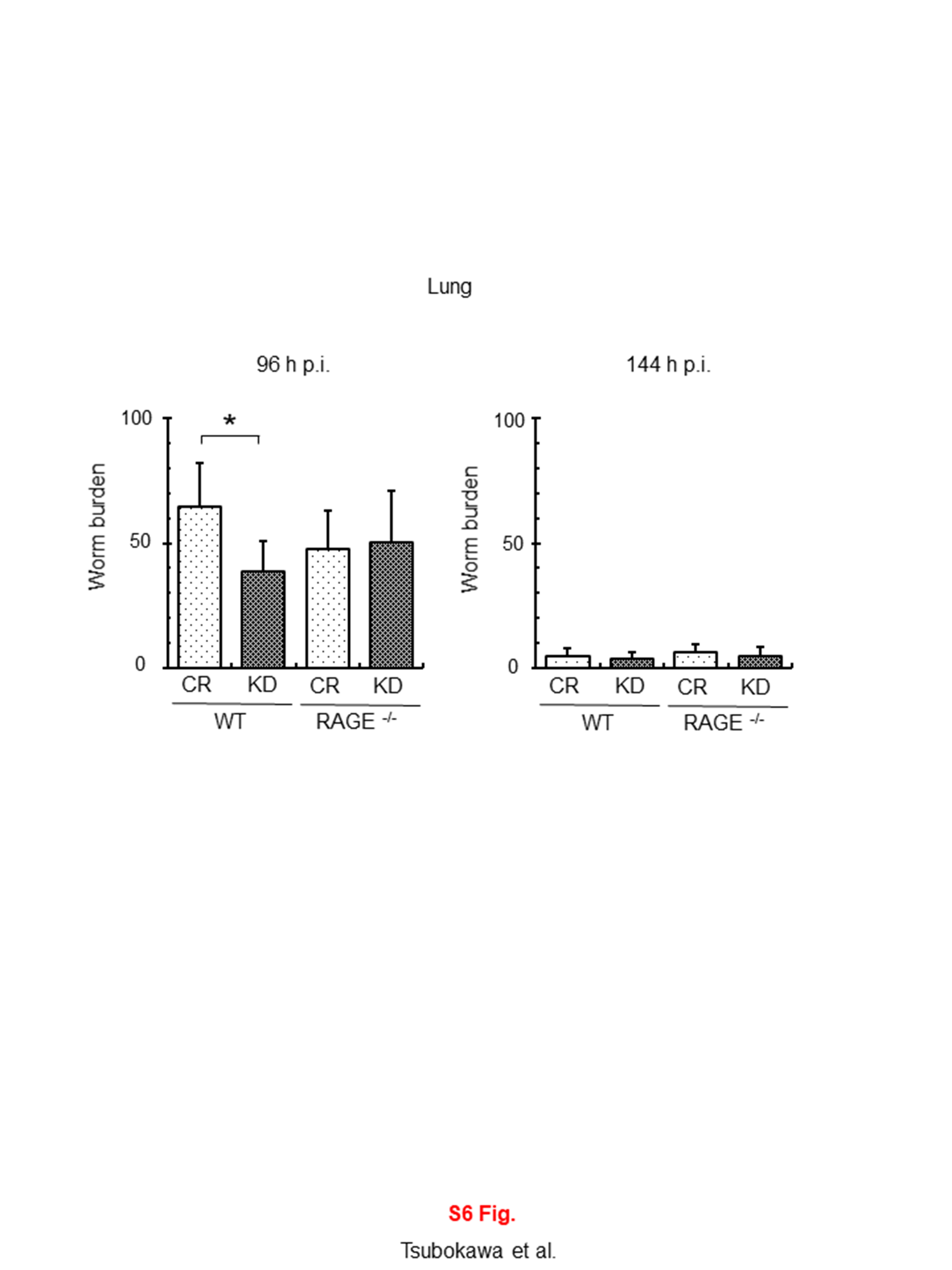

Supplement: S6 Fig — Wild-type (WT) or RAGE-null (RAGE-/-) mice were infected with 2,000 LL3s treated with dsluciferase (CR) or dsvenestatin (KD). Lung worm burdens from WT or RAGE-/- mice at days 4 (96 h) and 6 (144 h) p.i. are shown. Data are expressed as means ± SDs of 6 mice from two independent experiments. *p < 0.01. (TIF) [file ppat.1009649.s006.TIF]

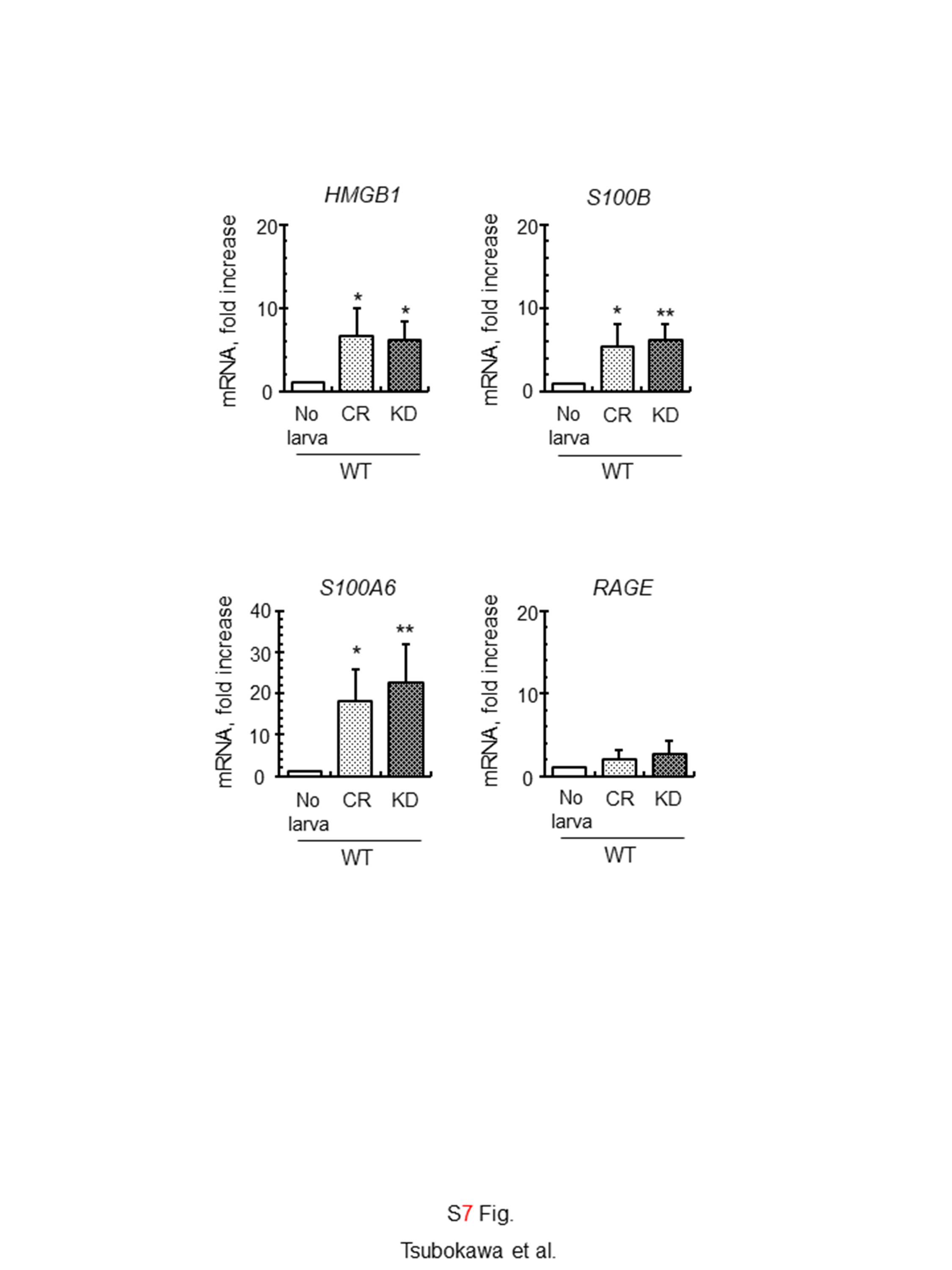

Supplement: S7 Fig — Quantitative RT-PCR analysis of RAGE and RAGE ligands (HMGB1, S100B, and S100A6) from skin tissue of wild-type (WT) mice was performed. Total RNA was extracted from mouse skin tissues at the larva inoculation site at 6 h p.i. with 2,000 LL3s treated with dsluciferase (CR) or dsvenestatin (KD). The mouse GADPH gene was used to normalize the amount of cDNA, and the expression level in naïve skin (no larva) was set as 1. Data are expressed as means ± SDs from three independent experiments with two technical replicates. *p < 0.01; **p < 0.001 from the no larva group. (TIF) [file ppat.1009649.s007.TIF]

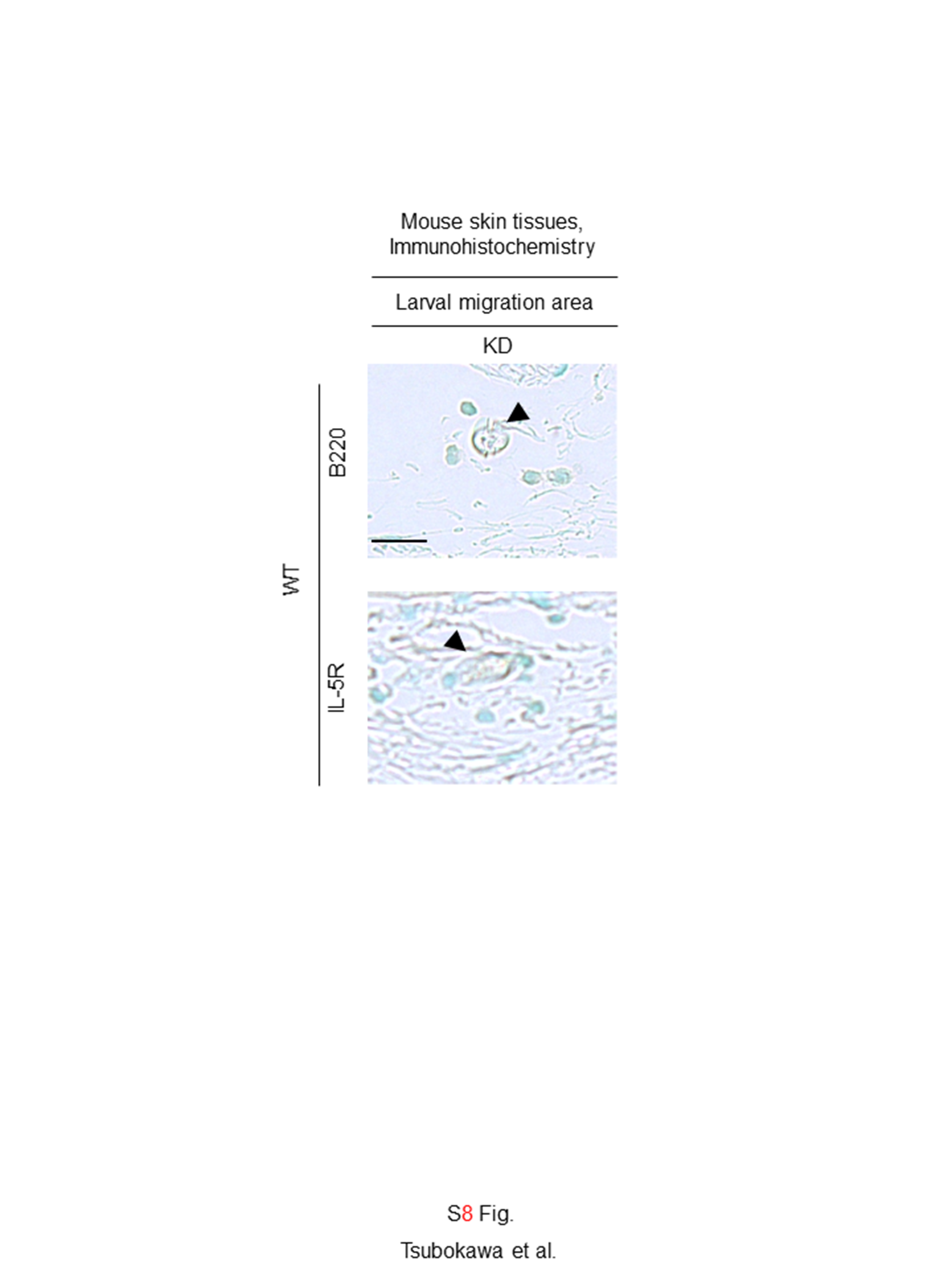

Supplement: S8 Fig — Immunohistochemical analysis of skin tissues from wild-type (WT) mice was performed. Skin tissues were collected from the larval inoculation site at 6 h p.i. with 2,000 LL3s treated with dsvenestatin (KD). The sections were subjected to immunostaining using anti-B220 (B cells) or anti-IL-5R (eosinophils) antibodies. Arrow heads show larval cross sections. Scale bar: 25 μm. (TIF) [file ppat.1009649.s008.TIF]

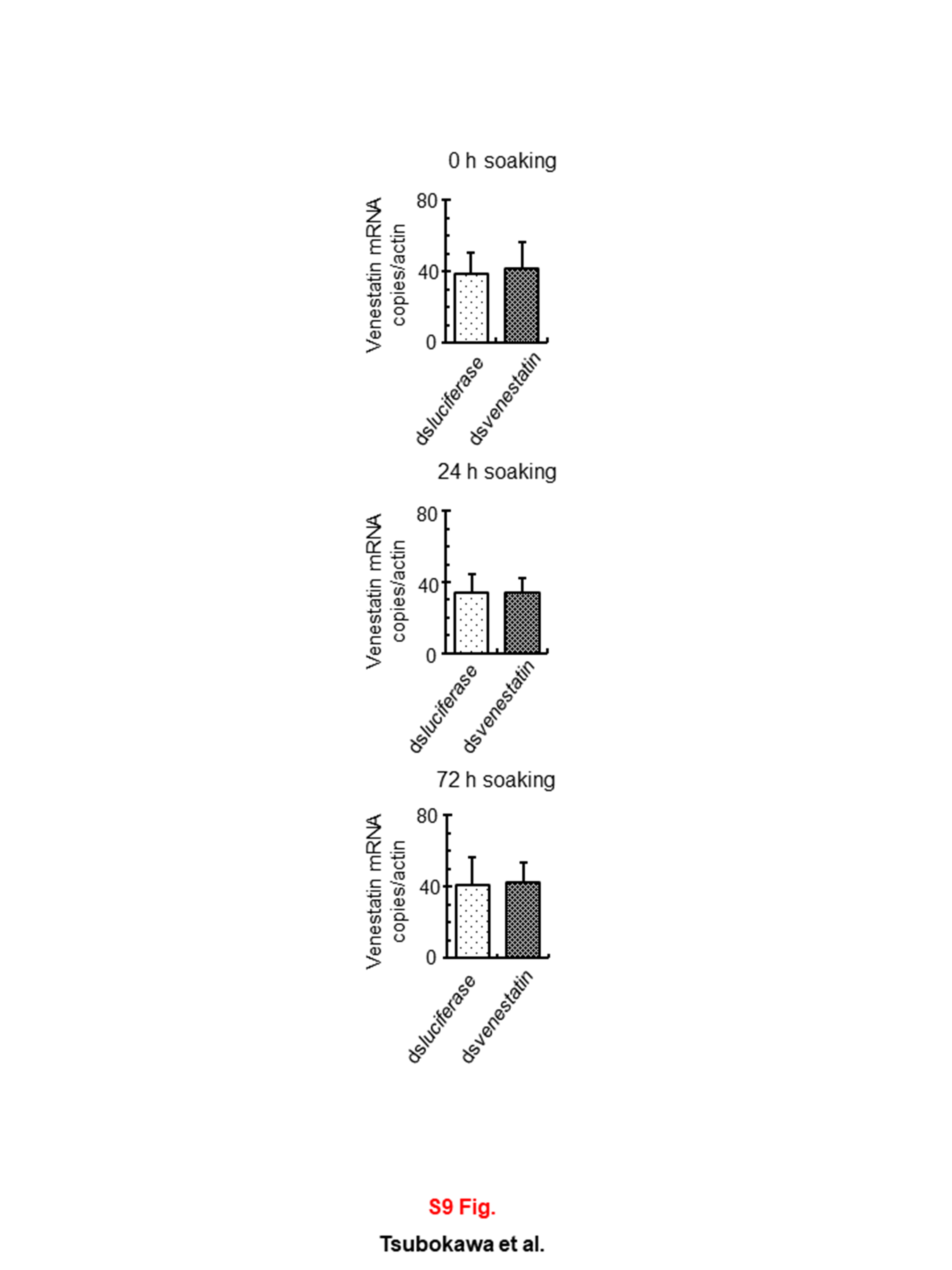

Supplement: S9 Fig — iL3s in the control group were incubated with luciferase dsRNA. The gene encoding S. venezuelensis actin-like protein (actin) was used as an internal control, and venestatin mRNA copies/actin was calculated. Data are expressed as means ± SDs for three independent experiments with two technical replicates. (TIF) [file ppat.1009649.s009.TIF]
