# Supplementary material for: Venestatin from parasitic helminths interferes with receptor for advanced glycation end products (RAGE)-mediated immune responses to promote larval migration
Source: PLoS Pathog. 2021 Jun 3;17(6):e1009649. doi: 10.1371/journal.ppat.1009649 (PMC8205142; doi:10.1371/journal.ppat.1009649)
Supplement: S1 Table — (DOCX) [file ppat.1009649.s010.docx]

**S1 Table. List of mouse PCR primers**

| **Gene** | **Forward primer** | **Reverse primer** |
| --- | --- | --- |
| *TNF-α* | acg gca tgg atc tca aag ac | cgg act ccg caa agt cta ag |
| *COX2* | aag ccg agc acc ttt gga g | att gat ggt ggc tgt ttt ggt ag |
| *IFN-γ* | cgg cac agt cat tga aag cct a | gtt gct gat ggc ctg att gtc |
| *IL-4* | cat cgg cat ttt gaa cga ggt ca | ctt atc gat gaa tcc agg cat cg |
| *IL-5* | aaa ttc ctg tag cgc agg ct | acc ctg atg caa cga aga gg |
| *IL-13* | agc tcc ctg gtt ctc tca ct | tcc tct ggg tcc tgt aga tg |
| *S100B* | agg aca gag gag ggg aat gg | ctc agc ctc acc aag ggc ta |
| *S100A6* | att cca tcc cct cg acca ct | tca gcc ttg caa ttt cag ca |
| *HMGB1* | gaa tgt gtc ttt aga tag ccc tgt cc | cgt ata agc tgc atc aga gac aac tg |
| *VCAM1* | gct cca gac att tac cca gtt tac ag | cct ggg aga gat gta gac ttg tag ttc |
| *eSelectin* | gtt gga gct cag aat cta cag tgt acc | gaa act ctg ctc aca ggt gaa gtt ac |
| *RAGE* | aga gcc act tgt gct aag ctg taa g | aat ctg gta gac tcg gac tcg gta gtt |
| *GADPH* | agc tga acg gga agc tca ct | tgg aag agt ggg agt tgc tg |
| *β-actin* | gct aca gct tca cca cca ca | aag gaa ggc tgg aaa aga gc |
