# Supplementary material for: Venestatin from parasitic helminths interferes with receptor for advanced glycation end products (RAGE)-mediated immune responses to promote larval migration
Source: PLoS Pathog. 2021 Jun 3;17(6):e1009649. doi: 10.1371/journal.ppat.1009649 (PMC8205142; doi:10.1371/journal.ppat.1009649)
Supplement: Attachment — Submitted filename: PPATHOGENS-D-21-00200 Responses to Reviewers2.doc [file ppat.1009649.s012.doc]

**Editor’s Comments**

**I believe there is concurrence among the reviewers that this paper constitutes a significant contribution to knowledge about immunomodulatory proteins elaborated by parasitic nematodes. Reviewer 3 was particularly effusive in his/her comments to the effect that this is an outstanding study of its kind. However, Reviewers 1 and 2 both bring up significant shortcomings in data gathered in existing experiments (eg. the need for additional controls) or, in the case of Reviewer 2, the need for some significant additional studies (cytokine profiles, worm migration kinetics and flow cytometric analysis of immune effector cells in inflammatory skin lesions) that should be carried out.**

**Response**: Thank you very much for your nice comments about our manuscript. We have tried our best to clarify the points raised by the reviewers. As suggested, we reexamined additional controls and newly assessed cytokine profiles, worm migration kinetics, and flow cytometry, which form an important part of the revised manuscript. In the revised version, we have added additional controls to the revised figures (**Revised Figures 1A and 1F**). We have also added examinations of cytokine profiles and worm migration kinetics. The pro-inflammatory cytokine was suppressed by venestatin in the RAGE-mediated inflammation models (**Supplementary Figure 3; Page 8, Lines 169-170**). Migration of some larvae from the skin was inhibited, but not delayed, by silencing of venestatin (**Supplementary Figure 6; Page 10, Lines 218-221; Pages 17-18, Lines 394-400**). These data support the conclusion in our manuscript. Although we tried to conduct flow cytometric analysis, technical problems hampered our ability to collect data (Response to Referee 2). In the present study, we concluded that venestatin suppressed RAGE-mediated immune responses in host skin induced by helminthic infection, promoting larval migration. We consider the identification of immune cells by flow cytometry unnecessary to support this conclusion.

**Response to the Reviewers’ Comments**

We appreciate the reviewer’s comments and their valuable suggestions.

**Referee 1**

We gratefully appreciate your kind and detailed comments and suggestions.

1. **Include RAGE only and Venestatin only controls for Fig. 1A**

**Response**: According to your suggestion, we have added RAGE only controls in Fig. 1A (white circles). Venestatin only controls were shown as the points labeled RAGE/TLR4 (0 μg/mL) (**Revised Figure 1A; Page 34, Lines 899-900**).

**2. Include Ca2+ chelating control experiments in Fig. 1F**

**Response**: Thank you for your valuable comment. We examined the reaction with EDTA as a Ca^2+^ chelating control. Some sentences describing this experiment have been added and the figure has been revised (**Revised Figure 1F; Page 22, lines 499-501; page 34, Lines 913-914**).

**3. Include details in text or supplemental data demonstrating how widespread venestatin is within the Phylum Nematoda**

**Response**: We previously reported a maximum likelihood tree of venestatin and selected nematode orthologs (Tsubokawa et al., Int J Parasitol 2017). Venestatin orthologs are widely distributed among nematode species. According to your suggestion, we have added a description of these points to the revised manuscript (**Page 13, Lines 290-294, 296-297**).

**4. More detailed discussion/interpretation is required for the RNAi specificity for LL3 versus iL3**

**Response**: According to your suggestion, we have added some discussion regarding RNAi specificity for LL3s over iL3s in the revised manuscript (**Pages 15-16, Lines 345-353**).

**5. Do the worm actin primers cross-react with mice. Please elaborate as it appears the host response was also used as an internal control. If they cross-react, how do the authors know which species they are monitoring?**

**Response**: The worm actin primers did not cross-react with mouse actin primer in this study, or vice versa. We have added a description of these points to the revised manuscript (**Figure 5A; Pages 10-11, Lines 229-232**).

**6. Fig 2A**: **add** **a magnified inset that marks examples of inflammatory cells. Also, are the white bars the PBS counts? this was unclear**

**Response:** According to your suggestion, we have added a magnified inset that shows examples of inflammatory cells in Fig. 2 (**Revised Figure 2; Page 35, Lines 921-922, 926-927**). Yes, the white bars represent the PBS control counts. To clarify this point, some sentences have been added to the figure legend (**Page 35, Lines 922-923, 927**).

**7. Fig. 3B should include loading controls.**

**Response**: As you pointed out, loading controls are important for quantitative evaluation of western blotting. Actin, GADPH, or tubulin, etc. in cells are useful as control proteins. However, we detected venestatin in ES products of larvae, including the culture media. In this case, the appropriate protein for use as a loading control was unknown. We have revised a sentence to clarify the alternative manipulation for quantitation in the revised manuscript (**Page 35, Lines 939-940**).

**8. Fig4B: RAGE mutant versus wt: are these significantly different? interpret? Also, the worm burden is still significant - how does this fit their model?**

**Response**: The worm burden did not show significant differences between WT mice and RAGE^-/-^ mice. We have added a sentence to clarify this point in the revised manuscript (**Page 10, Lines 213-214**).

**Minor Issues**

1. **Line 69: include reference(s) for secreted immunomodulators**

**Response**: According to your suggestion, we have added some references concerning secreted immunomodulators (**Page 4, Line 72**).

2. **Define HMGB1**

**Response**: As you pointed out, we have added a description of HMGB1 to the revised manuscript (**Page 4, Lines 84-85**).

**3. line 147: estimate "the" role**

**Response**: As you pointed out, we have edited this sentence in the revised manuscript (**Page 7, Line 152**).

**4. line 179: this sentence doesn't make sense. The authors state that soaking did not affect expression in iL3 and LL3s and conclude that soaking is specific to LL3s**
**Response**: As you mentioned, the intended meaning of this sentence was unclear. We meant that soaking with ds*venestatin*, but not ds*luciferase*, specifically disrupted venestatin mRNA. We have edited this sentence in the revised manuscript (**Pages 8-9, Lines 182-184**).

**5 line 233: authors switch to italics for proteins in contrast to non-italicized earlier. Be consistent.**
**Response**: According to your suggestion, we have applied consistent use of non-italics in the revised manuscript (**Pages 11-12, 25**).

**6. please change "rvenestatin" to "r-venestatin" or just "recombinant venestatin" to make it clearer for the reader**

**Response**: According to your suggestion, we have changed the terminology to “r-venestatin” in the revised manuscript.

**7. lines 438 and 445 and 449: insert space for new paragraph**

**Response**: According to your suggestion, we have inserted a space (**Page 21, Lines 477, 486, 492**).

**8. Include details of setting used with ClusPro 2.0**

**Response**: According to your suggestion, we have added the settings for running ClusPro 2.0 software (**Page 22, Lines 506-507**).

**9. line 138: Sequence similarity - include percentage**

**Response**: According to your suggestion, we have added the percentage (**Page 7, Line 141**).

**Referee 2**

We are very thankful for your kind suggestions and detailed comments.

**1. In the RAGE mediated inflammation models - both foot edema and pneumonia - only cellular infiltrates are examined. No examination of local or systemic pro-inflammatory cytokines or other mediators is examined. This needs to be performed.
Response**: Thank you for your valuable comment. According to your suggestion, we examined the cytokine profiles using sera of these mice in the revised manuscript (**Supplementary Figure 3; Page 24, Lines 561-563; Page 39, Lines 1028-1032**). Production of TNF-α, but not IFN-γ, was suppressed by venestatin in the RAGE-mediated inflammation models. The data supports the results of cellular infiltration (**Page 8, Lines 169-170**).

**2. In the experiments examining larval migration, kinetics of larval migration are not examined. While it is possible that migration is inhibited, it is also possible that migration is delayed and hence different time points post infection need to examined for worm burden etc.**

**Response**: We consider this to be an important point in the study. According to your suggestion, we examined the worm burden kinetics in the revised manuscript (**Supplementary Figure 6;** **Page 24, Line 544; Page 40, Lines 1045-1049**). The kinetics of larval migration to the lungs revealed that the worm burden in the lungs was decreased at 96 h p.i. compared with that at 72 h p.i. The larvae were hardly detected in the lungs of all groups at 168 p.i., regardless of venestatin expression (**Page 10, Lines 218-221**). Further, the worm burden of mature worms settling in the small intestines was reduced by silencing of venestatin (**Supplementary Figure 5**). These results indicated that migration of some of larvae from the skin was inhibited, but not delayed, by silencing of venestatin. We have added some sentences about this interpretation in the “Discussion” section of the revised manuscript (**Pages 17-18, Lines 394-400**).

**3. In terms of RAGE mediated inflammation of the skin, flow cytometry to examine different immune and non-immune cell types would be useful.**

**Response**: Thank you very much for your valuable comment. As you have pointed out, flow cytometry is useful for the detailed identification of immune cells. We attempted to perform flow cytometric analysis, but it was not possible to obtain sufficient cells from the infected tissues. The preparation of single cells for flow cytometry from skin, including subcutaneous tissues, is considered a technically challenging issue (Wetzels et al., J Vis Exp 2018; Sullivan et al., Front Vet Sci 2016). Further, obtaining cells from tissues during helminth infection is known to be difficult (Ferrer-Font et al., eLife 2020), requiring extensive examination to prepare single cells from nematode-invading tissues. In the present study, we wanted to emphasize the conclusion that venestatin suppresses RAGE-mediated inflammation in host skin induced by helminthic infection. Therefore, we believe that identification of specific immune cells is unnecessary to support this conclusion.

**Minor Issues**

**1. Typos in the manuscript need to be corrected.**

**Response**: We have checked and corrected typos in the revised manuscript (**Page 6, Line 116; Page 14, Line 324; Page 19, Line 428; Page 23, Line 531; Page 25, Line 581; Page 26, Line 606**).

**2. No discussion of the homologous proteins in either Strongyloides stercoralis or other lung migrating human helminths is done.**

**Response**: According to your suggestion, we have added a description of the venestatin homologs from *S. stercoralis,* *Necator americanus,* and *Schistosoma mansoni* (**Page 18, Lines 409-420**).

**3. What is the translational impact of this axis in human infections?**

Response: We speculate that elucidation of this axis will contribute to controlling cutaneous infection by human-infecting helminths. We have added a sentence to emphasize this point (**Pages 18, Lines 420-422**).

**Referee 3**

We are thankful for your kind suggestions and detailed comments.

**Minor Issues**

**1. The results presented in Figure 1A seem incomplete. Only 4 different concentrations are tested whereas the authors could perform a full curve that would allow them to calculate the strength of binding in the form of KD. Unless the protein is particularly difficult to produce, I can’t see a good reason to limit the data to what is included in the figure.**

**Response**: Thank you for your valuable comment. According to your suggestion,
we have added data for other concentrations in Fig. 1A, calculated the *K*_D_ value, and modified some sentences in the revised manuscript (**Revised Figure 1A; Page 6, Line 128; Page 20, Lines 461, 470; Page 34, Line 899**).

**2. Another aspect of the data that seemed incomplete is the lack of additional detail on the C2 domain. Figure 1D illustrates significant binding of venestatin to the C2 domain of RAGE (p<0.01), but this was not further explored. Was this binding also calcium-dependent? Including data for C2 in Figure 1F would be interesting and may add to the discussion point on the pathology induced by V domain interactions.**
**Response**: Thank you very much for your interest in our research. According to your suggestion, we examined the effects of Ca^2+^ on the binding of venestatin to the C2 domain. Although significant differences were not detected for the C2 domain, a Ca^2+^-dependent interaction trend was observed (**Figure 1G; Page 7, Lines 147-149; Page 34, Lines 911-912**). We have added some sentences in the “Discussion” section of the revised manuscript (**Page 14, Lines 315-318**).

**3. In reading the section on RNAi phenotypes (170-189), I wanted to see this data performed using iL3s. However, I understand the difficulty of RNAi and iL3s and it was helpful that the authors included a brief discussion of this, justifying their use of LL3s for these experiments. I agree with their use of LL3s and I do not think iL3s need to be used for this paper.**

**Response**: According to your suggestion, we have separated the LL3 and iL3 data (**Revised Figure 3A; Supplementary Figure 9; Page 15, Lines 344-345; Page 41, Lines 1067-1072**).

**4. Moving forward, the authors should consider activating the iL3s in vitro, which may facilitate RNAi in this life stage. Several labs use different techniques for activation of parasitic IJs. Morris et al. 2017 used the addition of serotonin to facilitate RNAi in Steinernema carpocapsae IJs. Hawdon et al. 1999 describes an activation technique for Ancylostoma caninum, but which has been used in other parasitic nematodes as well. Perhaps these methods would allow for efficient RNAi in S. venezuelensis iL3s.**

**Response**: We consider this to be crucial point. According to your suggestion, we have added some sentences with these references in the revised manuscript (**Pages 15-16, Lines 348-353**).

**5. There were a few minor textual issues as well. I did not catch all of them, but here are some suggested corrections:-Line 55, change to “downregulated…”
Line 274, change to “extracellular functions.”**

**Response**: We have corrected these words in the revised manuscript (**Page 3, Line 58; Page 13, Line 287**).
